# Aldo-ketoreductase 1c19 ablation does not affect insulin secretion in murine islets

**DOI:** 10.1371/journal.pone.0260526

**Published:** 2021-11-29

**Authors:** Yasutaka Miyachi, Taiyi Kuo, Jinsook Son, Domenico Accili

**Affiliations:** Department of Medicine and Naomi Berrie Diabetes Center, Vagelos College of Physicians & Surgeons of Columbia University, New York, New York, United States of America; International University of Health and Welfare School of Medicine, JAPAN

## Abstract

Beta cell failure is a critical feature of diabetes. It includes defects of insulin production, secretion, and altered numbers of hormone-producing cells. In previous work, we have shown that beta cell failure is mechanistically linked to loss of Foxo1 function. This loss of function likely results from increased Foxo1 protein degradation, due to hyperacetylation of Foxo1 from increased nutrient turnover. To understand the mechanisms of Foxo1-related beta cell failure, we performed genome-wide analyses of its target genes, and identified putative mediators of sub-phenotypes of cellular dysfunction. Chromatin immunoprecipitation analyses demonstrated a striking pattern of Foxo1 binding to the promoters of a cluster of aldo-ketoreductases on chromosome 13: Akr1c12, Akr1c13, Akr1c19. Of these, Akr1c19 has been reported as a marker of Pdx1-positive endodermal progenitor cells. Here we show that Akr1c19 expression is dramatically decreased in *db/db* islets. Thus, we investigated whether Akr1c19 is involved in beta cell function. We performed gain- and loss-of-function experiments in cultured beta cells and generated Akr1c19 knockout mice. We show that Foxo1 and HNF1a cooperatively regulate Akr1c19 expression. Nonetheless, functional characterization of Akr1c19 both using islets and knockout mice did not reveal abnormalities on glucose homeostasis. We conclude that reduced expression of Akr1c19 is not sufficient to affect islet function.

## Introduction

The pathogenesis of diabetes includes insulin resistance and pancreatic beta cell dysfunction [1]. There is a tight relationship between these two parameters, such that euglycemia can be maintained in insulin-resistant individuals throughout life, provided that insulin secretion remains robust [2]. Indeed, most insulin-resistant individuals do not develop diabetes. In contrast, impaired pancreatic beta cell function can result in hyperglycemia in individuals with modest levels of insulin resistance [3]. Moreover, whereas insulin resistance is relatively constant throughout an individual’s lifetime, beta cell function can deteriorate rapidly, especially in response to incipient hyperglycemia [4,5]. Thus, understanding beta cell failure is important to prevent, treat, and reverse diabetes [5].

Beta cell failure consists of multiple sub-phenotypes, whose contribution to the clinical condition likely differs in different patients [6]. It can be conveniently divided into defective insulin secretion and altered beta cell mass, although these two parameters at some level are integrated, and thus their alteration can reflect a common underlying process. Impaired insulin secretion is thought to precede decreased beta cell mass in the progression of diabetes [7]. Indeed, impaired insulin secretion in response to glucose can be demonstrated in individuals at risk prior to the clinical onset of diabetes [8]. In recent years, we have proposed a unifying mechanism for the two abnormalities, to the extent that they can be subsumed under the rubric of impaired FoxO1 function [9]. Thus, we have shown that progressive loss of FoxO1 function is found in genetic and acquired (e.g., diet) models of rodent diabetes, such as *db/db* mice and others [9]. When the FoxO1 deletion is brought about in a controlled fashion using gene knockouts, the resulting phenotypes recapitulate both the loss of insulin secretion [10], and the decrease of beta cell mass [9]. The latter we attributed to beta cell dedifferentiation, rather than death, a hypothesis borne out by studies of cadaveric human islets in diabetic patients from different ethnicities [11–13].

To deconvolute the pathogenesis of beta cell failure secondary to the observed progressive loss of FoxO1, we identified its target genes using a combination of chromatin immunoprecipitation and RNA profiling of beta cells isolated from diabetic mice [14–16]. From this analysis, we characterized selected candidates that regulate specific sub-phenotypes in the progression of beta cell dysfunction, including ALDH1A3 [16], the vitamin D binding protein Gc [15], the oxidoreductase Cyb5r3 [17], the co-regulator C2cd4a [14], and transcription factor Bach2 [16].

To catalog FoxO1 target genes in beta cells, we performed chromatin immunoprecipitation sequencing (ChIP-seq) in leptomycin B-treated islets from mice homozygous for FoxO1-GFP^Venus^ knock-in allele using a GFP antibody [14]. This approach allowed us to maximize the amount of nuclear FoxO1, and to increase the efficiency of immunoprecipitation using the more sensitive GFP antibody rather than commercial anti-FoxO1 antibodies. Because the fusion protein retains all the regulatory elements of the endogenous FoxO1 gene, expression levels match those of the wild-type FoxO1 allele [14]. Moreover, we have previously shown that the GFP tag does not interfere with FoxO1 trafficking and nuclear localization [18].

Our ChIP-seq analysis of FoxO1 in beta cells revealed an interesting pattern of DNA binding to three of the eight aldo-keto-reductase Akr1c genes clustered on chromosome 13 [19]. Aldo-keto-reductases are a group of NADPH-dependent oxidoreductase enzymes with different patterns of tissue distribution, comprised of three sub-families, Akr1, 6, and 7 [20]. Whereas Akr 6 and 7 are involved in the inactivation of voltage-gated K channels and aflatoxins, respectively [21,22], the Akr1 subfamily participates in the metabolism of steroid hormones and prostaglandins [20]. There are 9 genes encoding different Akr1c enzymes in mice, 8 of which are clustered on a 0.5 Mb region of chromosome 13. Given their role in hormone metabolism, we investigated whether they are *bona fide* FoxO1 targets and, if so, whether they affect beta cell function.

## Materials and methods

### Animals

C57BL/6J, leptin receptor deficient (*db*/*db*), and heterozygous Akr1c19 knockout (KO) mice on a C57BL/6NJ background were purchased from The Jackson Laboratory. Heterozygous KO mice were crossed to obtain homozygous KO mice and littermates (WT and heterozygous KO) were used as controls. All mice were maintained on a 12-h light and 12-h dark cycle with free access to normal chow diet (PicoLab rodent diet 20, 5053; Purina Mills) and water unless otherwise noted. At the end of the experiment, the animals were sacrificed by carbon dioxide inhalation. All animal experiments were in accordance with NIH guidelines for Animal Care and Use and approved by Columbia University Institutional Animal Care and Use Committee (protocol number AAAS9451).

### Cell culture

MIN6 and HEK293 cells were purchased from AddexBio Technologies and ATCC, respectively. MIN6 cells were maintained in DMEM with 4.5 g/L glucose and L-glutamine without sodium pyruvate (Corning) containing 15% fetal bovine serum (FBS), penicillin-streptomycin and 0.05 mM 2-mercaptoethanol. For gene expression analysis, MIN6 cells were transduced with adenovirus encoding dominant negative FoxO1 and GFP at an MOI of 500 and incubated for 48 h. Cells were also treated with FoxO1 inhibitor (AS18420856, 100 nM) and incubated for 24 h. HEK293 cells were maintained in DMEM with 1.0 g/L glucose and L-glutamine without sodium pyruvate (Corning) containing 10% FBS and penicillin-streptomycin. Islets were isolated by collagenase digestion as previously described [17]. Isolated islets were incubated overnight in RPMI 1640 containing 10% FBS with penicillin-streptomycin and used for further analyses.

### Metabolic analyses

Blood glucose and serum insulin concentrations were measured with OneTouch glucose monitors (One Touch Ultra, Bayer) and ELISA (Mercodia), respectively. For glucose tolerance tests, mice were fasted for 16 h with free access to water followed by intraperitoneal glucose injection (2 g/kg). We measured blood glucose at 0, 15, 30, 60, 120 min after injection. For sulfonylurea challenge test, random-fed mice were injected intraperitoneally with glibenclamide (5 mg/kg). We measured blood glucose and insulin levels at 0, 5, 15, 30 min after injection.

### Plasmids

FoxO1 and dominant negative FoxO1 (D256) expression plasmids were described before [23]. HNF1a, MafA, and Pdx1 expression plasmids were purchased from OriGene. Akr1c19 promoter regions were amplified by PCR from genomic DNA isolated from the liver of an 8-week-old male C57BL/6J and subcloned into pGL4.10 vector (Promega) using EcoRV with an In-Fushion cloning kit (Takara Bio). The forward primers -1022/-1, -621/-1, -401/-1, -264/-1, and -139/-1 (numbered relative to ATG) Akr1c19 promoter regions were 5’- GCTAGCCTCGAGGATAAATATGAGTACCACTACTGTACAC -3’, 5’- GCTAGCCTCGAGGATACTGAGGGAAATATAGGTAAGAGAC -3’, 5’- GCTAGCCTCGAGGATAGGTTTCTATCTAGAAAACAACCAG -3’, 5’- GCTAGCCTCGAGGATATCTGAATGATACCAAGTCACAGG -3’, and 5’- GCTAGCCTCGAGGATATCATTAATCAGATAACAACACTGC -3’, respectively. The reverse primer Akr1c19 promoter regions was 5’- AGGCCAGATCTTGATTGCTTATCACTCTTCTGACTAAGC -3’.

### Luciferase promoter assay

HEK293 and MIN6 cells were transfected with 200 ng of luciferase reporter construct and total 10 ng of plasmids using Lipofectamine 3000 transfection reagents in 24-well plates. Red fluorescent protein (RFP) was used as a negative control. After 48 h incubation, cells were lysed and the luciferase activity was measured using Dual-Luciferase reporter assay system (Promega). Renilla luciferase was used as an internal control. For promoter deletion assay, MIN6 cells were treated with FoxO1 inhibitor, AS18420856 (100 nM) after 24 h of transfection and further incubated for 24 h.

### Immunohistochemistry

Pancreata were fixed in 4% paraformaldehyde (PFA) at room temperature for 2 h, placed in 30% sucrose at 4°C for 2 days and embedded in optimal cutting temperature (OCT) compound. Frozen sections (7μm) were air-dried and boiled in a microwave oven for 1 min in Tris-EDTA buffer (pH 9.0). Anti-Akr1c19 antibody (sc-393680, 1:100 dilution; Santa Cruz Biotech) was used to detect Akr1c19-positive cells. Anti-glucagon antibody (G2654, 1:100 dilution; Sigma Aldrich) and anti-insulin antibody (ab7842, 1:50 dilution; Abcam) were used to detect alpha cells and beta cells, respectively. Glucagon- and insulin-positive areas were analyzed using ImageJ.

### RNA isolation and quantitative PCR

RNA was isolated with RNeasy Mini-kit (Qiagen) and reverse-transcribed using qScript cDNA SuperMix (Quanta). Quantitative real-time PCR was performed using GoTaq qPCR Master Mix (Promega). We analyzed data with the ΔΔCT method. Primers for Akr1c19 were: (F: 5’- GAAATGTTGCTGGTCCAAGG -3’, R: 5’- TGGATCATTCAAGAGAACTGGA -3’). Other Primer sequences are available on request.

### Protein analysis

MIN6 cells were lysed and fractionated into cytoplasm, membrane, and nucleus (9038, Cell Signaling Tech) according to the manufacturer’s instruction. Isolated islets were lysed using RIPA buffer (89900, Thermo Scientific). Samples were separated by 10% SDS-PAGE and transferred to PVDF membranes. Immunoblotting was performed using anti-Akr1c19 (sc-393680, 1:1000 dilution; Santa Cruz Biotech), anti-MEK1/2 (8727, 1:1,000 dilution; Cell Signaling Tech), anti-AIF (5318, 1:1,000 dilution; Cell Signaling Tech), anti-Histone H3 (4499, 1:1,000 dilution; Cell Signaling Tech), and anti-GAPDH (ab181602, 1:10,000 dilution; Abcam) antibodies. Uncropped blot images are shown in Supporting Information (S4 Fig).

### Statistical analyses

All data were analyzed using Graph Pad Prism 8 and were presented as mean ± standard error of mean. A *p* value < 0.05 was considered statistically significant. Unpaired t-test was used to compare two groups. One-way ANOVA followed by Dunnett’s multiple comparison was used for comparison between three groups. Two-way ANOVA followed by Sidak’s multiple comparison test was used to examine the influence of two different variables.

## Results

### Identification of Akr1c19 and its regulation by FoxO1 and HNF1a

**Fig 1:**
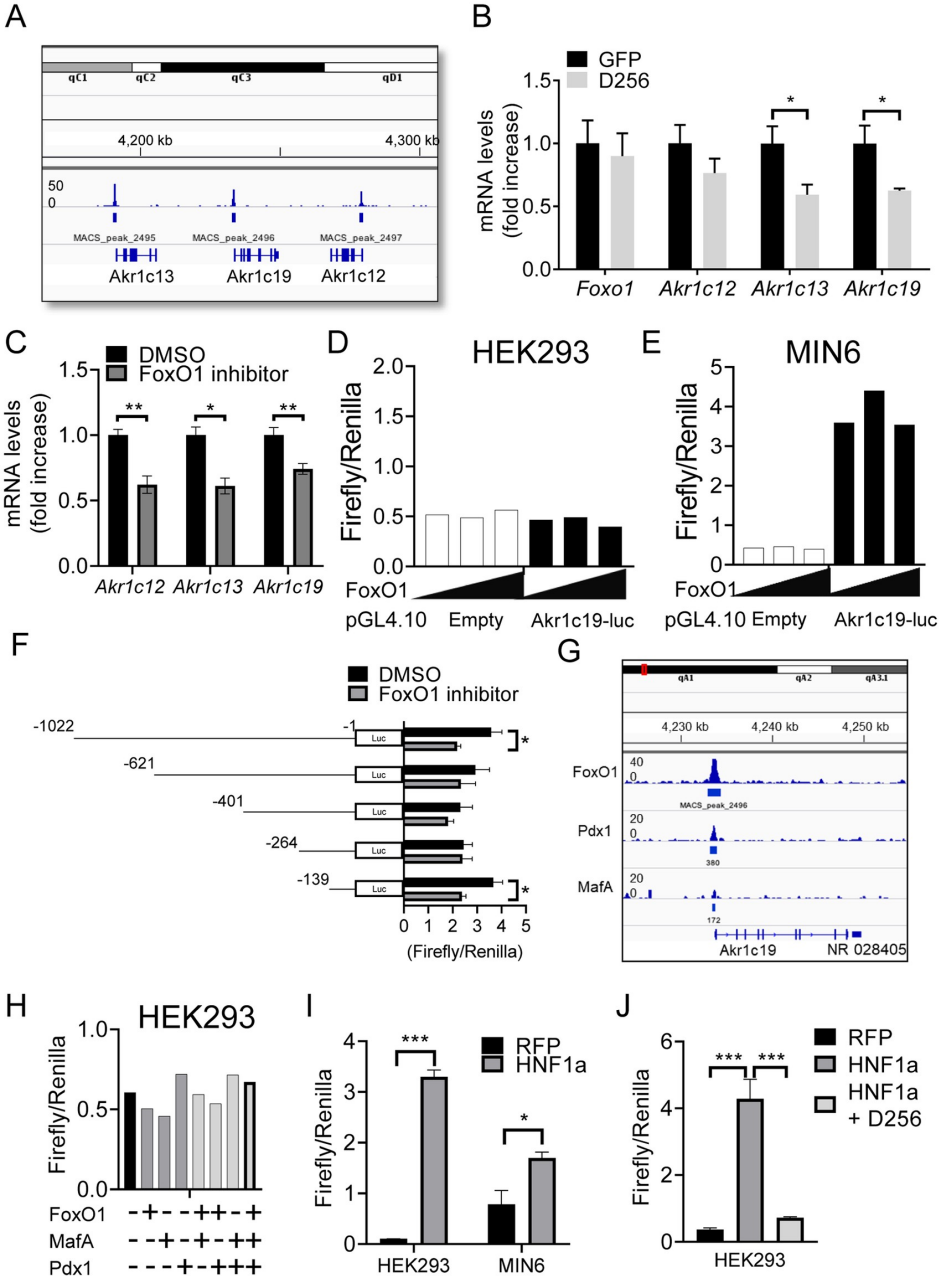
Akr1c19 promoter analysis. (A) FoxO1 ChIP-seq in leptomycin B-treated mouse islets. Akr1c12, Akr1c13, and Akr1c19 loci are shown. The y-axis indicates tag counts. (B-C) Gene expression analysis in MIN6 cells (B) transduced with adenovirus encoding dominant negative FoxO1 (D256) and GFP and (C) after treatment with the FoxO1 inhibitor, AS18420856 (100 nM). (D-E) Akr1c19 promoter activity after co-transfection of different amounts of FoxO1 (0, 1, 10 ng) and pGL4.10, containing 1,022 bp upstream of TSS in the Akr1c19 promoter region in (D) HEK293 and (E) MIN6 cells. (F) Effect of the FoxO1 inhibitor AS18420856 (100 nM) on Akr1c19 promoter activity in MIN6 cells transduced with pGL4.10 containing 1,022, 621, 401, 264, or 139 bp upstream from of Akr1c19 TSS. (G) Integrated ChIP-seq analysis of FoxO1, MafA, and Pdx1 in Akr1c19 locus visualized in integrative genomics viewer. (H) Akr1c19 promoter activity in HEK293 cells overexpressing various combination of FoxO1, MafA, and Pdx1. (I-J) Akr1c19 promoter activity (I) in HEK293 and MIN6 cells overexpressing HNF1a and (J) in HEK293 cells overexpressing HNF1a and D256. All values represent mean ± SEM. * *p* < 0.05, ** *p* < 0.01, *** *p* < 0.001 by Student’s t-test and ANOVA.

We identified FoxO1 binding sites within 1kb from the transcription start site (TSS) of three adjacent Akr1c genes on chromosome 13: Akr1c12, Akr1c13, and Akr1c19 (Fig 1A). To test whether they are regulated by FoxO1, we performed loss-of-function experiments in MIN6 cells by transducing them with adenovirus encoding dominant negative FoxO1. We observed a ~50% decrease in the levels of Akr1c13 and c19 isoforms, and no changes to Akr1c12 (Fig 1B). Given that the efficiency of adenoviral transduction was ~75%, sought to achieve a more extensive decrease using a chemical FoxO1 inhibitor, AS18420856 [24]. Treatment with this compound reduced expression of all three isoforms to an extent comparable to the dominant negative FoxO1 in MIN6 cells (Fig 1C). Although the function of three Akr1c genes in beta cells remains unknown, Akr1c19 [25] was reported to be a marker of ES-cell-derived definitive endoderm and to be co-expressed with insulin in the pancreas at E14.5 [26]. We thus focused further studies on this isoform.

To investigate the mechanism by which FoxO1 regulates Akr1c19, we performed luciferase reporter assays. We co-transfected increased amount of FoxO1 expression plasmid with a fixed amount of Akr1c19 luciferase reporter plasmid containing up to 1,022 bp upstream from TSS of Akr1c19 promoter region into HEK293 and MIN6 cells. This region spans the FoxO1 binding site identified by ChIP-seq. However, FoxO1 did not induce Akr1c19 promoter activity in either cell type (Fig 1D and 1E), whereas we detected reporter activity in MIN6 cells in the absence of FoxO1 (Fig 1E). These data indicate that FoxO1 alone is not sufficient to induce Akr1c19 promoter activity.

To determine whether FoxO1 is necessary to regulate Akr1c19 reporter activity, we performed promoter deletion analyses by transfecting MIN6 cells with luciferase constructs bearing deletions of the Akr1c19 promoter, and measuring the latter in the absence or presence of AS18420856. We detected a ~40% decrease of Akr1c19 promoter activity in MIN6 cells transfected with constructs –1,022 bp and –139 bp following incubation with AS18420856 (Fig 1F). In contrast, the inhibitor did not affect reporter activity in cells transfected with constructs bearing deletions of –621 bp, –401 bp, and –264 bp (Fig 1F).

Since FoxO1 alone had little effect on Akr1c19 promoter activity, we investigated whether additional transcription factors are required to regulate this process. To this end, we interrogated publicly available ChIP-seq data for Pdx1, NeuroD, FoxA2, and MafA sites in the Akr1c19 promoter. We found that Pdx1 and MafA peaks overlap with the FoxO1 peak in the Akr1c19 promoter, consistent with regulation of this gene by important beta cell transcription factors (Fig 1G). We tested Pdx1 and MafA, alone or in combination with each other and with FoxO1, in Akr1c19 reporter assays using HEK293 cells. However, neither Pdx1 nor MafA transfection affected reporter gene activity, regardless of the combination (Fig 1H).

Inspection of the promoter sequence revealed several HNF1a consensus cis-acting DNA elements. Therefore we tested the role of HNF1a, one of maturity onset diabetes of the young (MODY) genes [27]. Transfection of HNF1a in HEK293 or MIN6 cells increased Akr1c19 promoter activity by ~30- and 2-fold, respectively (Fig 1I), showing a role of HNF1a to regulate Akr1c19 expression. In contrast to Akr1c19, we did not observe HNF1a-induced promoter activation of Akr1c12 and 13 in HEK293 cells (S1 Fig). In previous work, we have shown that the three FoxO genes (1, 3a, and 4) are required to maintain the MODY-related gene network [10,28]. Therefore, to investigate the involvement of FoxO1 in the regulation of Akr1c19 by HNF1a, we co-expressed HNF1a and a dominant negative FoxO1 D256, a truncated mutant lacking the transactivation domain [23], in HEK293 cells. D256-FoxO1 inhibited HNF1a-induced Akr1c19 promoter activity back to basal levels (Fig 1J), indicating that Foxo1 is required for HNF1a-mediated Akr1c19 gene expression.

### Expression and localization of Akr1c19 in diabetic mouse islets

**Fig 2:**
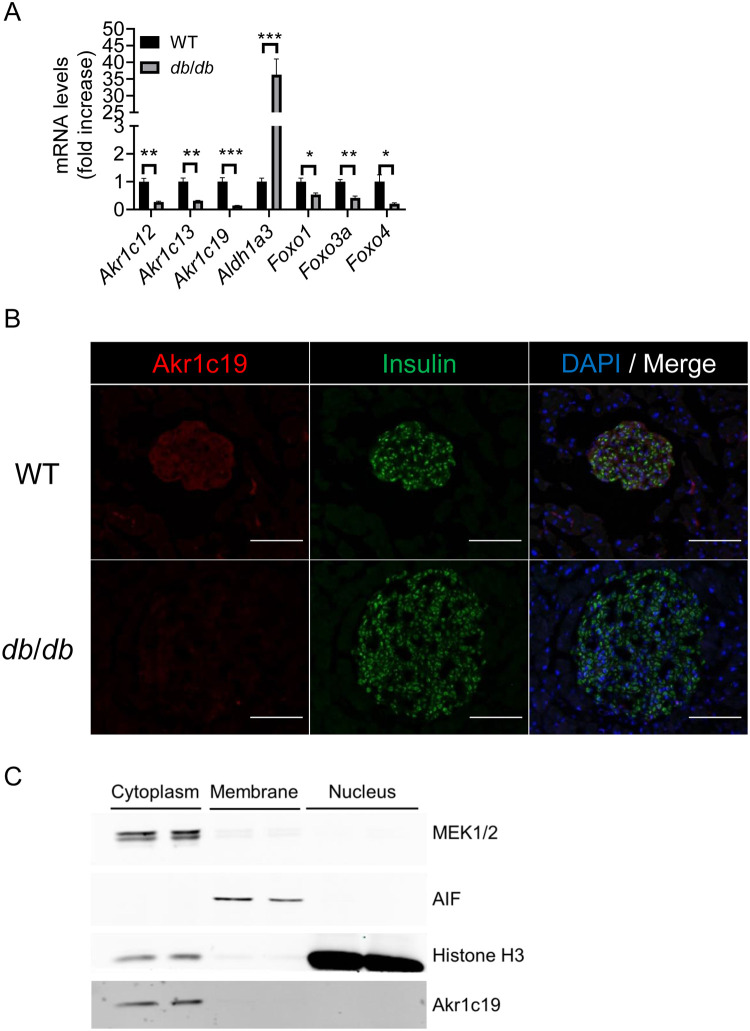
Akr1c19 expression in *db*/*db* islets and localization in MIN6 cells. (A) Gene expression analysis in islets from 12-week-old WT and *db*/*db* male mice (n = 4, each group). (B) Immunostaining for Akr1c19 (red), insulin (green), and DAPI (blue) in islets from WT (upper) and *db*/*db* (lower). Scale bar = 100 μm. (C) Immunoblotting for Akr1c19 in subcellular fractions of MIN6 cells. All values represent mean ± SEM. * *p* < 0.05, ** *p* < 0.01, *** *p* < 0.001 by Student’s t-test.

To understand whether Akr1c19 affects beta cell function, we examined expression of the three Akr1c genes in islets of diabetic mice. Analysis of islets isolated from 12-week-old *db*/*db* mice revealed that expression of Akr1c12, Akr1c13, and Akr1c19 decreased, as did that of FoxO1, FoxO3a, and FoxO4. In contrast, consistent with previous findings, Aldh1a3, a marker of beta cell failure, showed a 40-fold increase (Fig 2A) [10]. Immunostaining of pancreata from wild-type mice demonstrated that the vast majority of Akr1c19-expressing cells co-expressed insulin (Fig 2B). Consistent with the mRNA expression data, levels of Akr1c19 were nearly undetectable in insulin-immunoreactive cells from *db*/*db* islets (Fig 2B). Since *db/db* mice have beta cell dysfunction, these findings are consistent with the possibility that Akr1c19 is involved in the process.

To determine the localization of Akr1c19 in beta cells, we performed subcellular fractionation of MIN6 cells into membrane, cytosolic, and nuclear fractions, and tested their purity by probing with compartment-specific markers. Akr1c19 expression was restricted to the cytoplasmic fraction (Fig 2C), consistent with an intracellular enzymatic function.

### Generation and analysis of Akr1c19 knockout mice

**Fig 3:**
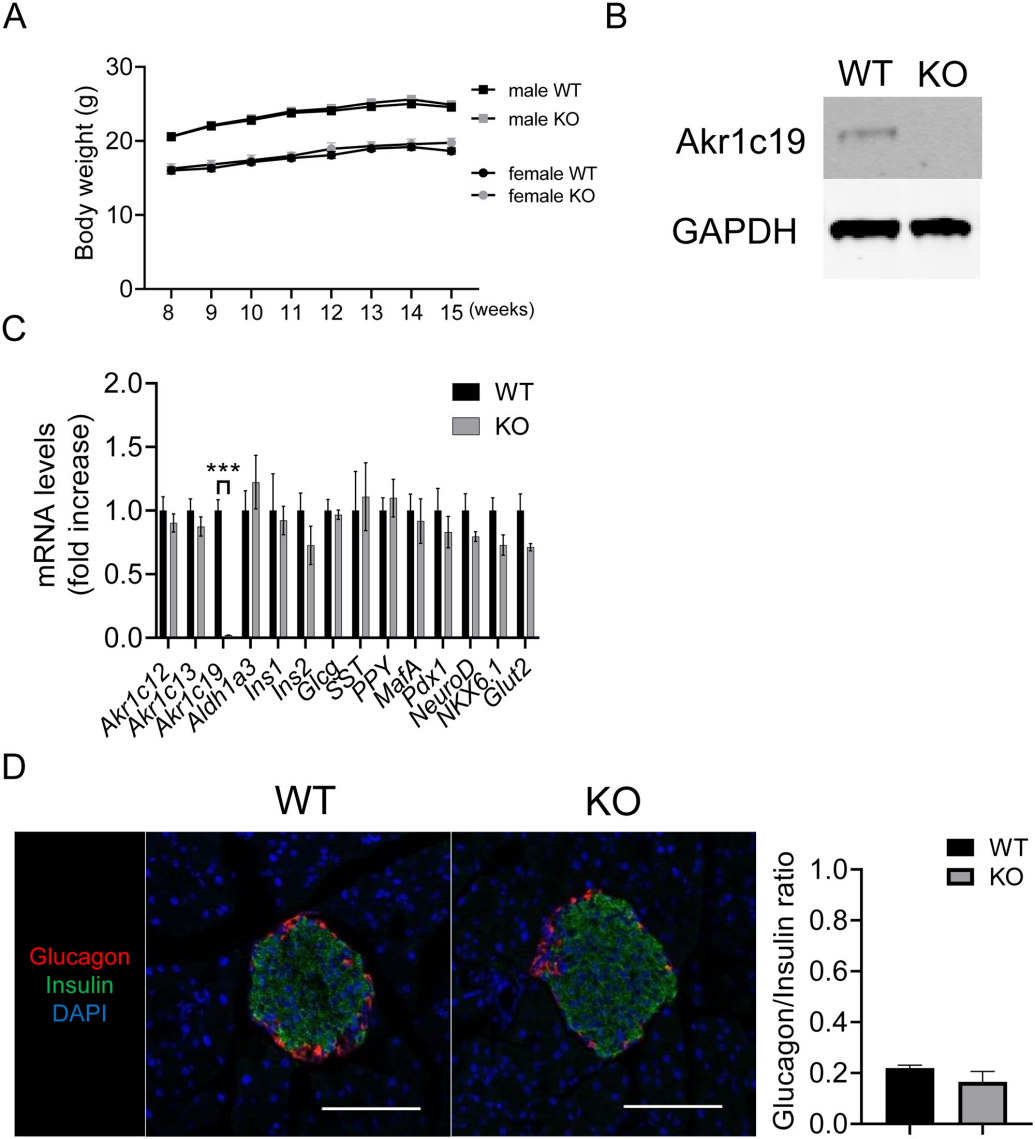
Development and islet morphology of Akr1c19 knockout mice. (A) Changes in body weight in of male and female Akr1c19 knockout (KO) mice. (B) Immunoblotting for Akr1c19 and (C) gene expression analysis in isolated islets from WT and KO mice. (D) Immunostaining for glucagon (red), insulin (green), and DAPI (blue) in WT and KO islets and quantification of glucagon/insulin ratio. Scale bar = 100 μm. All values represent mean ± SEM. *** *p* < 0.001 by Student’s t-test.

We generated a constitutive ubiquitous knockout of *Akr1c19* (KO) in mice to determine the physiological role of Akr1c19. KO mice were born at the expected Mendelian frequency and developed normally (Fig 3A). We confirmed deletion of Akr1c19 in islets isolated from KO mice, with a ~98% reduction of Akr1c19 mRNA (Fig 3B and 3C). Gene expression analysis of isolated islets showed no difference in alpha, beta, delta, and PP cell markers between the two genotypes (Fig 3C). We further characterized the distribution and abundance of glucagon- and insulin-expressing cells in pancreata from KO mice to assess whether Akr1c19 is involved in the establishment of proper islet morphology and cell mass. There were no changes in islet morphology and glucagon/insulin ratio between the two genotypes (Fig 3D). Moreover, glucose-stimulated insulin secretion (GSIS) in KO islets was comparable to that in WT islets (S2 Fig).

**Fig 4:**
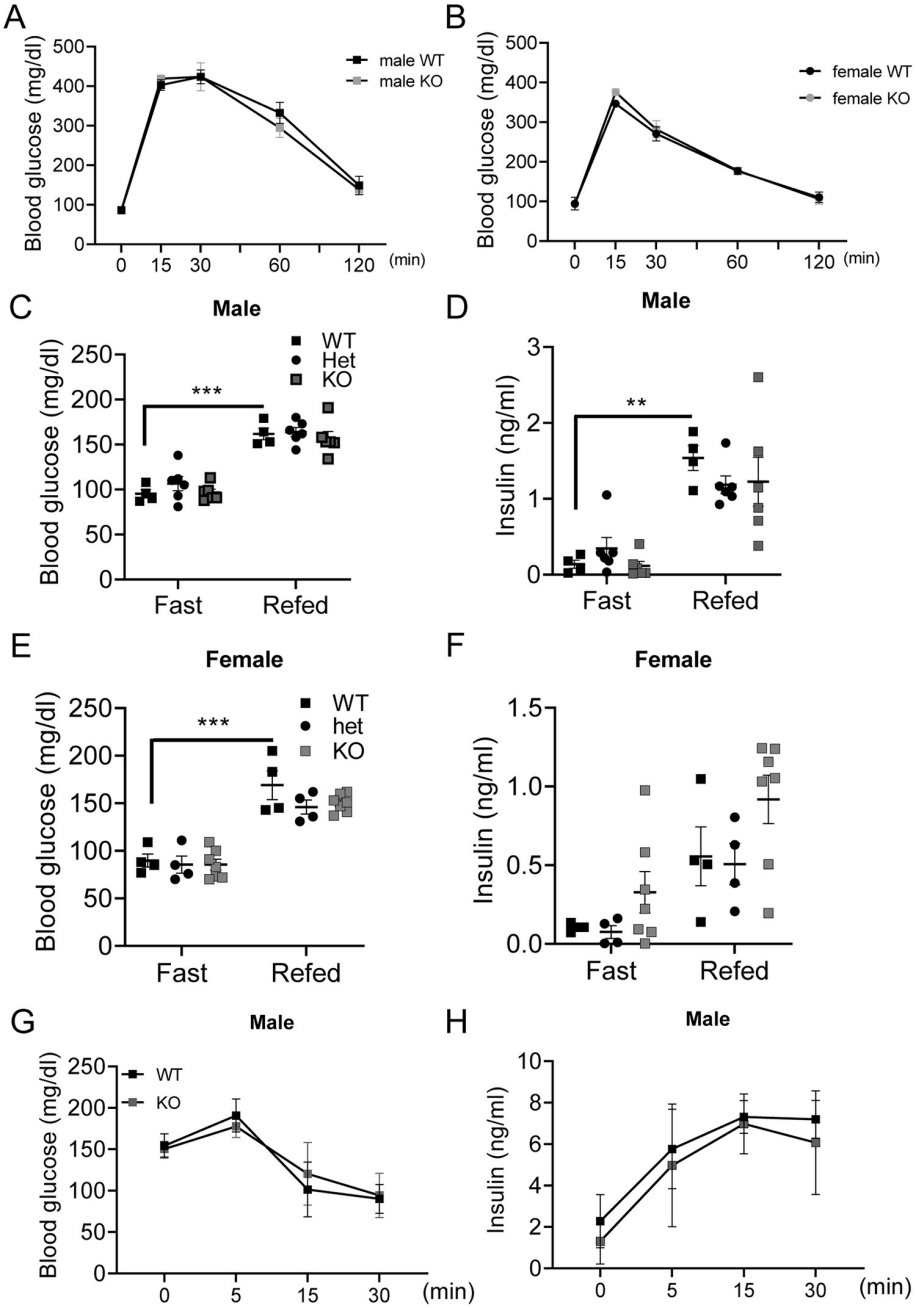
Whole body glucose metabolism and insulin secretion in KO mice. (A-B) Glucose tolerance tests in 16-week-old (A) male and (B) female KO mice. Male WT (n = 4), male KO (n = 6), female WT (n = 4), and female KO (n = 6). (C-F) Glucose and insulin concentrations in heterozygous (Het) and homozygous KO mice fasted overnight (Fast) or fasted overnight and then refed for 1h (Refed). (C) Blood glucose and (D) insulin levels in male mice. Male WT (n = 4), male Het (n = 6), and male KO (n = 6). (E) Blood glucose and (F) insulin levels in female mice. Female WT (n = 4), female Het (n = 4), and female KO (n = 7). (G) Blood glucose and (H) insulin levels in male KO mice after intraperitoneal injection of glibenclamide (5 mg/kg). WT (n = 5), and KO (n = 3). All values represent mean ± SEM. ** *p* < 0.01, *** *p* < 0.001 by ANOVA.

To determine the role of Akr1c19 on glucose metabolism *in vivo*, we performed intraperitoneal glucose tolerance tests in 16-week-old KO mice. Akr1c19 deficiency did not affect glucose tolerance in male and female mice (Fig 4A and 4B). Furthermore, there were no differences in glucose tolerance at the age of 32 or 48 weeks (S3 Fig). Next, we assessed glucose and insulin levels in heterozygous and homozygous KO male mice after overnight fasting followed by 1h-refeeding. Glucose and insulin levels were normal in both genotypes (Fig 4C and 4D). We observed similar results in female mice (Fig 4E and 4F). Crystal structure analysis has shown that human AKR1Cs contain binding sites with sulfonylurea drugs, and that sulfonylureas interact with AKR1Cs [29]. Therefore, we examined whether there are changes to sulfonylurea-induced insulin secretion. To this end, we measured glucose and insulin levels in KO mice after intraperitoneal injection of glibenclamide. KO mice showed a normal response to glibenclamide challenge (Fig 4G and 4H).

## Discussion

We have been systematically mapping the FoxO1 regulome in beta cells in order to describe the overall process of beta cell dedifferentiation. During our analyses, we were struck that three members of the Akr1c gene cluster on chromosome 13 showed distinct FoxO1 DNA binding peaks. As this family of enzymes has been implicated in hormone catabolism, and given the striking decrease of their expression in *db/db* islets, we decided to investigate its function in beta cells. We focused on the one isoform that had been implicated in pancreatic development, and for which reagents were readily available, Akr1c19. We show here that its regulation by FoxO1 is indirect, and is mediated by way of Hnf1a. This functional interaction is consistent with the role ascribed to FoxO1 to control the MODY gene network [10], and may occur in a similar fashion to the FoxO1/Hnf4a interaction [30–32]. Despite suggestive evidence of involvement of Akr1c19 in beta cell function, such as the striking decrease in *db/db* islets, we were unable to detect an effect on beta cell mass, differentiation, or insulin secretion in Akr1c19 KO mice. This may be due to compensation by additional Akr1c isoforms, because in addition to our finding that FoxO1 binds to the promoter of three Akr1c isoforms, the three Akr1c isoforms also consist of 323 amino acids, and the amino acid sequence of Akr1c19 is about 73% identical to that of Akr1c12 and 13. Unfortunately, due to their co-localization within a 0.5Mb of chromosome 13, the genes are too close to generate combined knockouts by conventional breeding strategies, and too far apart to do so by CRISPR or other homologous recombination approaches. It may well be that only a complete loss of Akr1c function can affect beta cell function, and that the residual Akr1c activity in the Akr1c19 knockouts protects them from failure. This is a limitation of the present study.

There was a strong rationale behind our choice of Akr1c genes as effectors of beta cell failure. Akr1c12, Akr1c13, and Akr1c19 are decreased in islets of *db/db* mice, while ALDH1A3 is markedly elevated. We have previously reported the association between ALDH1A3 and all-*trans*- and 9-*cis*-retinoic acid (RA) in islets of *db/db* mice [16]. ALDH1A3 synthesizes RA from retinaldehyde, and it has been reported that retinaldehyde is reduced to retinol by Akr1c [33]. Therefore, the reduction of Akr1c12, Akr1c13, and Akr1c19 might have further facilitated the utilization of retinaldehyde for RA production of by ALDH1A3 in *db*/*db* islets. Considering the role of RA in pancreatic development [34], the hypothesis that Akr1c19 was involved in RA metabolism seemed attractive. However, given that the islets of KO mice are normal, Akr1c19 alone likely doesn’t affect RA metabolism.

The failure to find a phenotype in Akr1c19 knockouts stands in contrast to previous analyses of additional FoxO1 target genes. In our original publication on this topic, we had emphasized that the repertoire of FoxO1 targets altered during beta cell failure is relatively narrow [10]. However, each one of the targets functionally tested so far appears to be involved in a different sub-phenotypes of beta cell failure. For example the vitamin D-binding protein Gc is associated with beta- to alpha-cell transition, as predicted from both rodent and human studies [11,15], whereas Cyb5r3 is associated with impaired mitochondrial complex III function [17], and Bach2 with actual dedifferentiation [35]. In conclusion, this work demonstrated that isolated loss-of-function of Akr1c19 is not sufficient to alter beta cell function. The availability of this information and the reagents developed for this study may be of interest to other investigators and prevent a duplication of similar efforts, or be used in conjunction with other reagents to address the role of this family of enzymes in a collective fashion.

## Supporting information

S1 Fig HNF1a-induced promoter activities of Akr1c12 and 13 in HEK293 cells. HEK293 cells were co-transfected with HNF1a and luciferase reporter construct containing Akr1c12 (-1115 to -1) or Akr1c13 (-1006 to -1) promoter region. All values represent mean ± SEM.
(PDF)

S2 Fig Glucose-stimulated insulin secretion in isolated islets from KO mice. Isolated islets from 10-week-old female KO mice were incubated in 100 ul Kreb’s buffer (118.5 mM NaCl, 1.19 mM KH2PO4, 1.19 mM MgSO4, 10 mM HEPES, 2% BSA, with 2.54 mM CaCl2) with low glucose (1.0 g/L) or high glucose (4.5 g/L) for 1h. Supernatants of 5 islets were collected to measure insulin levels. Female WT (n = 4), and female KO (n = 4). All values represent mean ± SEM. * *p* < 0.05 by two-way ANOVA with *post-hoc* Tukey tests.
(PDF)

S3 Fig Glucose tolerance tests in KO mice. Glucose tolerance tests in 32-week-old male and 48-week-old female KO mice. Male WT (n = 4), male KO (n = 6), female WT (n = 4), and female KO (n = 6). All values represent mean ± SEM.
(PDF)

S4 Fig Uncropped Western blot images. (PDF)
