# Supplementary figures and images for: Aldo-ketoreductase 1c19 ablation does not affect insulin secretion in murine islets
Source: PLoS One. 2021 Nov 29;16(11):e0260526. doi: 10.1371/journal.pone.0260526 (PMC8629236; doi:10.1371/journal.pone.0260526)

S1 Fig

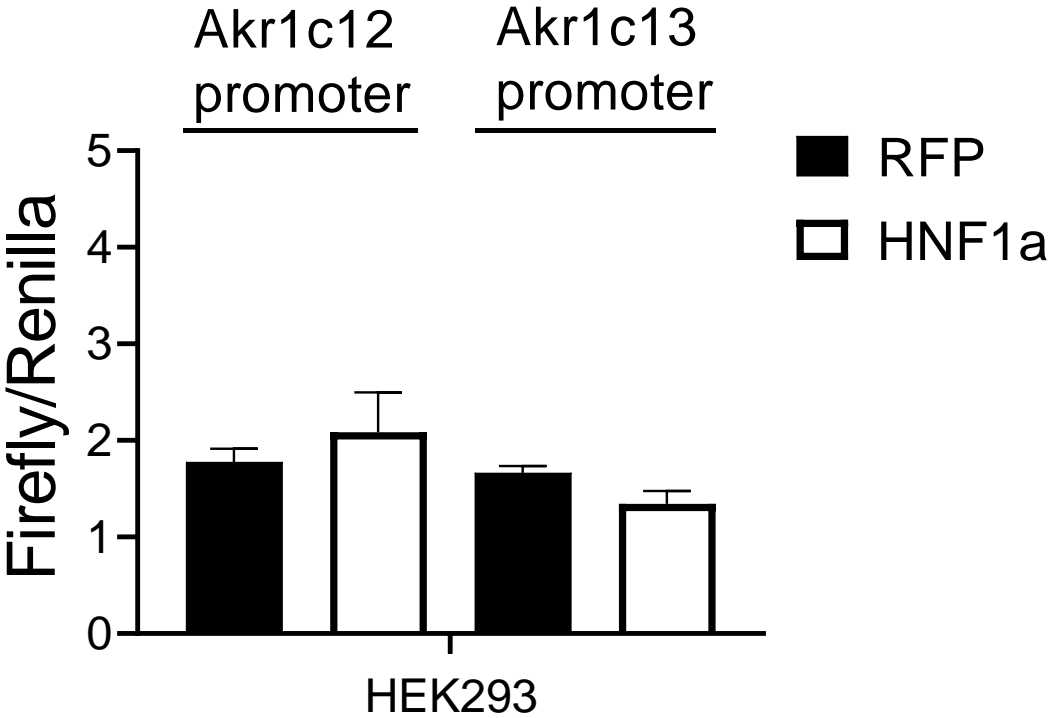

Supplement: S1 Fig — HEK293 cells were co-transfected with HNF1a and luciferase reporter construct containing Akr1c12 (-1115 to -1) or Akr1c13 (-1006 to -1) promoter region. All values represent mean ± SEM. (PDF) [file pone.0260526.s001.pdf]

S2 Fig

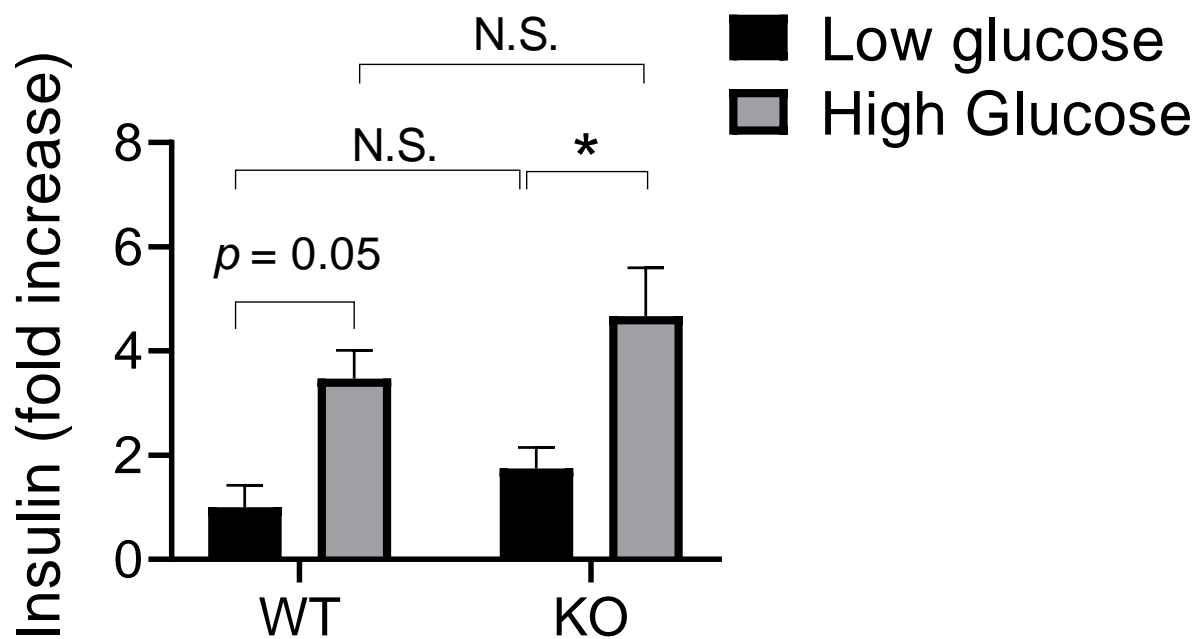

Supplement: S2 Fig — Isolated islets from 10-week-old female KO mice were incubated in 100 ul Kreb’s buffer (118.5 mM NaCl, 1.19 mM KH2PO4, 1.19 mM MgSO4, 10 mM HEPES, 2% BSA, with 2.54 mM CaCl2) with low glucose (1.0 g/L) or high glucose (4.5 g/L) for 1h. Supernatants of 5 islets were collected to measure insulin levels. Female WT (n = 4), and female KO (n = 4). All values represent mean ± SEM. * p < 0.05 by two-way ANOVA with post-hoc Tukey tests. (PDF) [file pone.0260526.s002.PDF]

S3 Fig

32-week-old

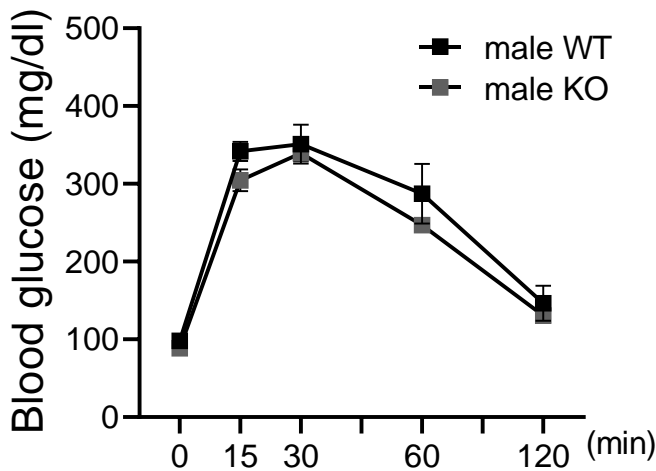

48-week-old

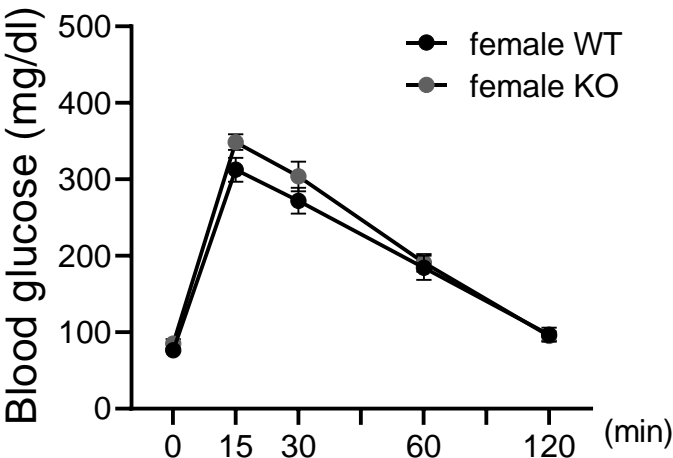

Supplement: S3 Fig — Glucose tolerance tests in 32-week-old male and 48-week-old female KO mice. Male WT (n = 4), male KO (n = 6), female WT (n = 4), and female KO (n = 6). All values represent mean ± SEM. (PDF) [file pone.0260526.s003.pdf]
