# Supplementary material for: Aldo-ketoreductase 1c19 ablation does not affect insulin secretion in murine islets
Source: PLoS One. 2021 Nov 29;16(11):e0260526. doi: 10.1371/journal.pone.0260526 (PMC8629236; doi:10.1371/journal.pone.0260526)

S4 Fig

Related to Fig.2C

Akr1c19 (36kDa)

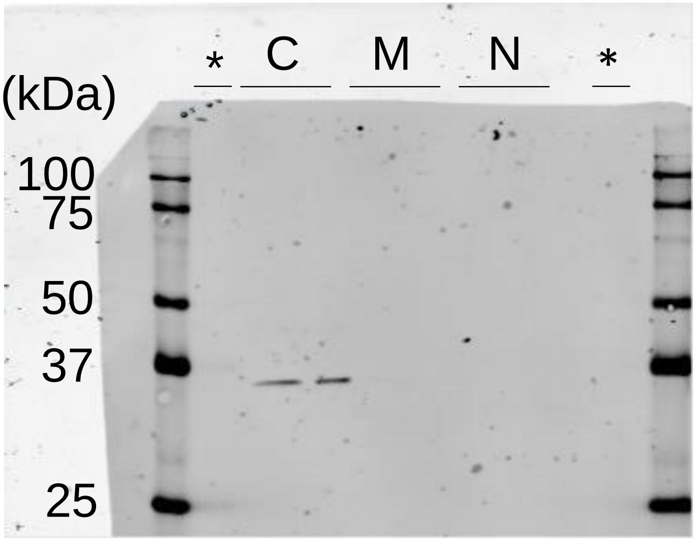

MEK1/2 (45kDa)

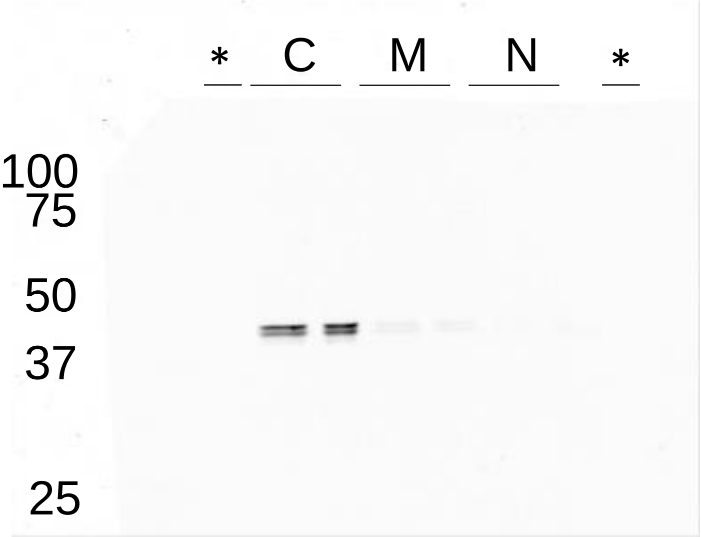

AIF (67kDa)
Histone H3 (17kDa)

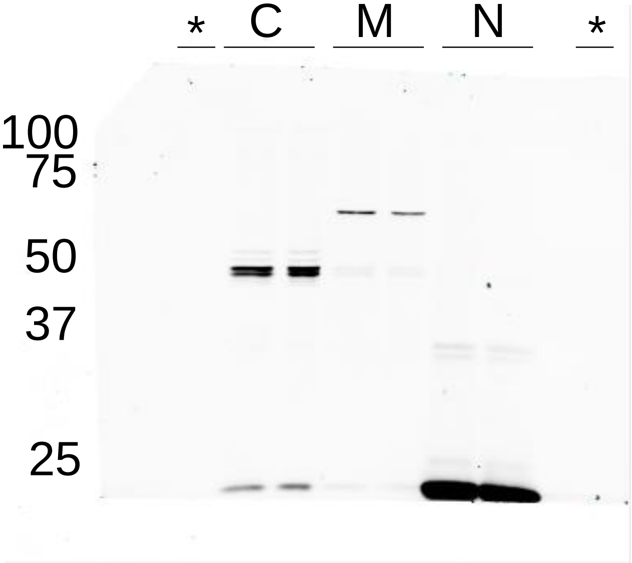

* Blank

C: Cytoplasm

M: Membrane

N: Nucleus

Related to Fig.3B

Akr1c19 (36kDa)

All samples are islets:

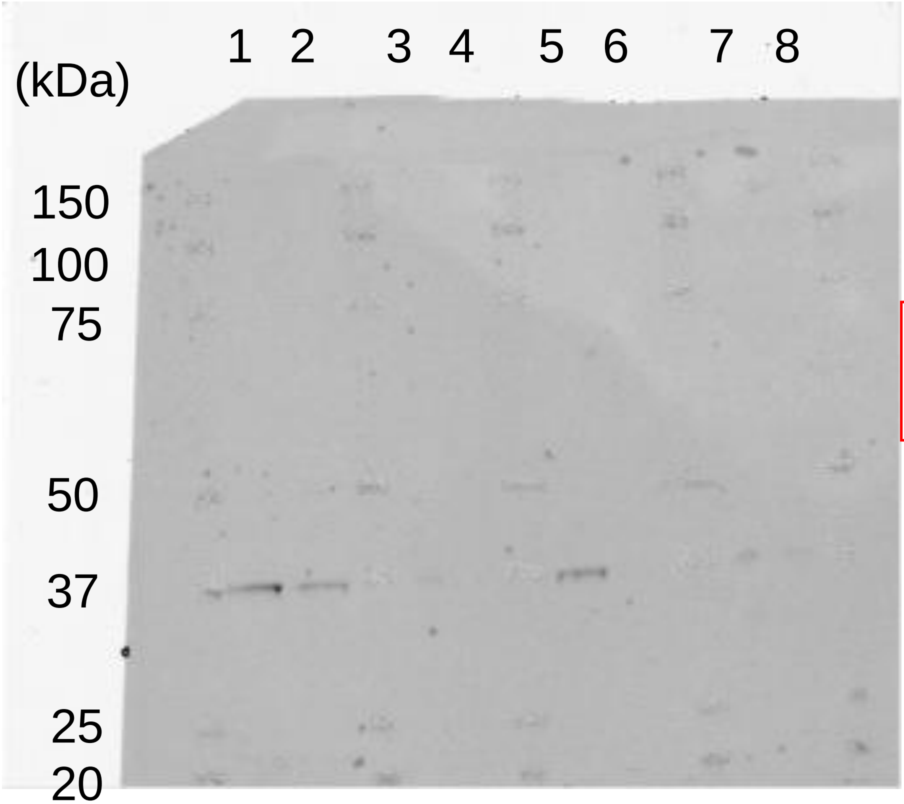

1 WT
2 *db/db*
3 WT
4 *db/db*
5 WT
6 Akr1c19 KO
7 *db/db*
8 *db/db*

GAPDH (37kDa)

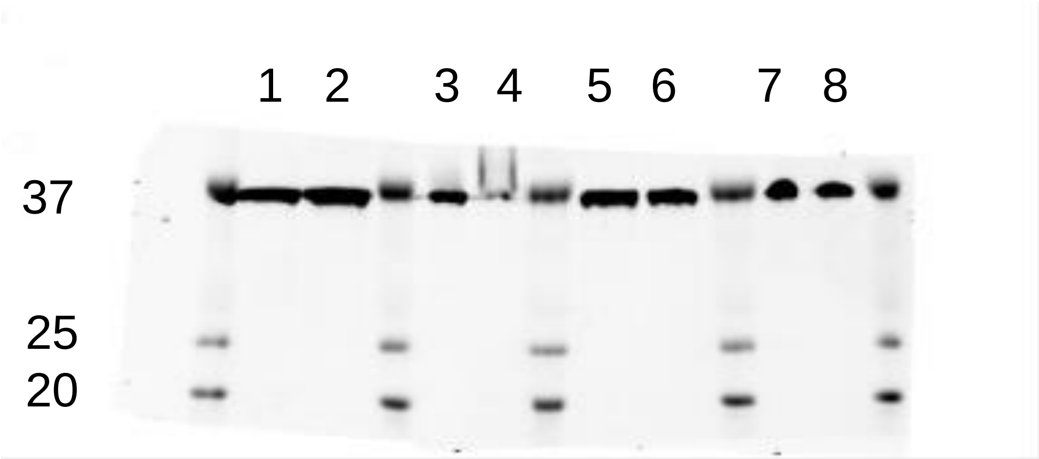

Supplement: S4 Fig — (PDF) [file pone.0260526.s004.pdf]
